# HAMP as a Prognostic Biomarker for Colorectal Cancer Based on Tumor Microenvironment Analysis

**DOI:** 10.3389/fonc.2022.884474

**Published:** 2022-08-05

**Authors:** Fang-Ze Wei, Shi-Wen Mei, Zhi-Jie Wang, Jia-Nan Chen, Fu-Qiang Zhao, Juan- Li, Ti-Xian Xiao, Wei Zhao, Yun-Bin Ma, Wei Yuan, Qian Liu

**Affiliations:** ^1^ Department of Colorectal Surgery, National Cancer Center/National Clinical Research Center for Cancer/Cancer Hospital, Chinese Academy of Medical Sciences and Peking Union Medical College, Beijing, China; ^2^ State Key Laboratory of Molecular Oncology, National Cancer Center/National Clinical Research Center for Cancer/Cancer Hospital, Chinese Academy of Medical Sciences and Peking Union Medical College, Beijing, China

**Keywords:** CRC, biomarker, HAMP, TME, immune

## Abstract

Colorectal cancer (CRC) is the most common digestive tumor in the world and has a high mortality rate. The development and treatment of CRC are related to the immune microenvironment, but immune response-related prognostic biomarkers are lacking. In this study, we used The Cancer Genome Atlas (TCGA) to explore the tumor microenvironment (TME) and weighted gene coexpression network analysis (WGCNA) to identify significant prognostic genes. We also identified differentially expressed genes in the TCGA data and explored immune-related genes and transcription factors (TFs). Then, we built a TF regulatory network and performed a comprehensive prognostic analysis of an lncRNA-associated competitive endogenous RNA network (ceRNA network) to build a prognostic model. CCR8 and HAMP were identified both in the WGCNA key module and as immune-related genes. HAMP had good prognostic value for CRC and was highly expressed in CRC tissues and had a negative correlation with CD4^+^ T cells and M0 macrophages based on immunohistochemistry and immunofluorescence staining of clinical specimens.We found that HAMP had high prognostic and therapeutic target value for CRC and was associated with liver metastasis. These analysis results revealed that HAMP may be a candidate immune-related prognostic biomarker for CRC.

## Introduction

Colorectal cancer (CRC) is the third most common digestive cancer and the second deadliest cancer worldwide (1). CRC accounts for approximately 10% of all new cancer cases globally. The increase in the incidence of and mortality associated with CRC is due to the lack of diagnostic biomarkers and the lack of an understanding of the occurrence mechanism (2). Given the development of CRC therapy, there are now more choices for primary and metastatic patients; however, there are still some limitations. Increasing evidence has shown that the tumor microenvironment (TME) greatly influences the development of CRC (3) and that the TME plays an important role in tumor development. The coaction between tumor cells and their supporting cells and changes in metabolism and the immune environment have a close relationship with tumor growth and development (4).

Therefore, the TME significantly influences the clinical therapeutic response and prognosis of cancer patients. Some studies have reported the influence of stromal cells on tumor angiogenesis and extracellular matrix remodeling, but the genetic alterations and mechanism remain largely unexplored (5, 6). Recently, the immune system has been considered to play an important role in tumor development, and tumor-infiltrating immune cells (TICs) in the TME serve as a key indicator of the therapeutic effects in and survival of CRC patients (7–10).

The diagnosis and monitoring of CRC occurrence and progression are dependent on examinations, including endoscopic surveillance and serum biomarker measurements (11); however, these methods have some limitations. Some patients with the same TNM classification have different clinical outcomes, and in some patients, the serum biomarker levels do not change (12). Therefore, it is necessary to identify biomarkers that are associated with the TME and easy to monitor.

In this study, we explored immune-related differentially expressed genes (IRDEGs) between CRC tissue and normal tissue based on The Cancer Genome Atlas (TCGA) database. After screening by univariate Cox analysis, we identified prognostic immune-related differentially expressed genes (PIRDEGs), constructed a regulatory network of transcription factors (TFs) and performed a comprehensive prognostic analysis of an lncRNA-associated competitive endogenous RNA network (ceRNA network). Based on the differentially expressed genes (DEGs), we built an immune prognostic risk score model. We used the ESTIMATE and CIBERSORT methods to calculate the tumor-infiltrating lymphocytes of the immune and stromal components of the CRC samples from the TCGA database. After that, we identified a predictive biomarker through weighted gene coexpression network analysis (WGCNA). Based on the two prognostic DEGs, we identified HAMP, which is a key gene that regulates iron metabolism. HAMP was highly expressed in CRC tissue, and we evaluated its expression in CRC cells and tissues. We continued to explore the potential biological value of HAMP and found that HAMP had a negative correlation with CD4^+^ T cells and M0 macrophages based on the results of immunohistochemistry and immunofluorescence staining of clinical specimens. In addition, we used R packages to explore the functions of these genes in immunity and performed gene set enrichment analysis (GSEA) to investigate their potential functions in CRC.

## Materials and Methods

### Gene Expression Datasets and CRC Clinical Samples

All microarray datasets were downloaded from the TCGA database. RNA data were downloaded from the TCGA database (https://portal.gdc.cancer.gov/), including 41 control tissues and 421 CRC tissues with clinical data. The GSE14297 dataset was downloaded from the GEO database (http://www.ncbi.nlm.nih.gov/geo). Sixty-eight surgical samples, including tumor tissue and pericarcinomatous tissues, were obtained from patients who were clinically diagnosed at our cancer hospital from October 2020 to June 2021. Samples were collected after approval by the ethics committee of Cancer Hospital Institute.

### Identification of Significant IRDEGs

Based on the mRNA profiles in the TCGA database, we used the “limma” and “edgeR” R packages to identify DEGs (13) between tumor and normal tissues following the criteria |log (fold change)| >1 and P value <0.05. Then, we downloaded immune-related genes from the IMMPORT database (https://www.immport.org/) (14) and explored the IRDEGs that overlapped both the DEGs in the TCGA database and the immune-related genes.

### Development of a TF Regulatory Network and a ceRNA Network

Then, we downloaded TFs from the Cistrome database (http://cistrome.org/) (15) and identified the genes overlapping TF genes and prognostic immune-related genes screened by univariate analysis. After that, we explored the relationship between IRDEGs and TF-DEGs and constructed a TF-IRDEG regulatory network by using Cytoscape software (version 3.6.1). Based on the miRNA and lncRNA matrix files of CRC downloaded from the TCGA database, we identified differentially expressed miRNAs (DEmiRNAs) and lncRNAs (DElncRNAs) with the R package “edgeR” with P<0.01 and |logFc|≥2 as cutoffs. Based on the prognostic genes identified by multivariate Cox proportional hazards analysis, we identified candidate mRNAs as targets of the DEmiRNAs that were recognized by three databases (TargetScan, miRTarBase, and miRDB) and explored the interactions between the DElncRNAs and DEmiRNAs. Based on the interactive regulatory relationships of the DEmiRNAs and DElncRNAs and the DEmiRNAs and DEmRNAs, we utilized Cytoscape software 3.6.1 to construct an lncRNA−miRNA−mRNA−ceRNA network.

### Development of a Prognostic Model and Immune Infiltration and Risk Scores

We performed univariate and multivariate Cox proportional hazards analyses to screen the IRDEGs to identify hub IRDEGs with prognostic value and built a prognostic risk score prediction model using the “survival” R package and SangerBox online tool. We validated the model through receiver operating characteristic (ROC) curve analysis. We used CIBERSORT to explore the relative immune cell percentages in the hub genes in all tumor tissues (16). Immune information was downloaded from TIMER (17) (https://cistrome.shinyapps.io/timer/) to explore the correlation between the risk score and immune infiltration score through the R packages “limma”, “reshape2”, “tidyverse” and “ggplot2”. In addition, we utilized the online tool SangerBox to explore the relationship between the hub IRDEGs.

### TME Component Score and Identification of Significant DEGs

We used the “ESTIMATE” and “limma” R packages to estimate the proportions of structural components (stromal and immune cells) in the TME. Then, we calculated the immune score, stromal score, and ESTIMATE score of tumor tissue, divided the three scores into high- and low-score groups, and plotted the survival curves of each group with the “survminer” and “survival” R packages. We used CIBERSORT to explore the relative percentage of immune cells in CRC tumor tissue and used immunohistochemistry to validate the expression on CD4^+^ T cells and M0 macrophages. The antibodies used to detect these immune cells were CD4 rabbit mAb (48274S) and CD68 XP rabbit mAb (76437S), which were purchased from CST. The immunohistochemistry process is shown in the **Supplemental Materials and Methods**.

We used the “limma” R package to explore the DEGs between the high- and low-score groups based on the immune score and stromal score with the following criteria: |log (fold change)| >1 and P value <0.05. Then, we used the “VennDiagram” R package to identify genes shared in terms of the immune score and stromal score. After obtaining the intersecting DEGs, we performed WGCNA with the “WGCNA” R package to identify clinical trait-related modules (18).

### GO and KEGG Functional Enrichment Analyses

We conducted Gene Ontology (GO) and Kyoto Encyclopedia of Genes and Genomes (KEGG) pathway enrichment analyses using the “org.Hs.e.g.db”, “enrichplot”, “ggplot2”, and “GOplot” R packages, and an adjusted P value of <0.05 was considered statistically significant.

### Immune Hub Genes Associated With the TME and Prognostic Value

We used the “VennDiagram” R package to identify immune hub genes associated with the TME and explored their prognostic value through the Gene Expression Profiling Interactive Analysis (GEPIA) (http://gepia.cancer-pku.cn/) online tool (19). We used the GEO online tool LOGpc (http://bioinfo.henu.edu.cn/DatabaseList.jsp) (20) to explore the protein expression and prognostic value of HAMP. The LOGpc online prognostic tool encompasses 209 expression datasets and provides 13 different survival terms for 31310 patients with 27 distinct malignancies.

### Expression of HAMP and Its Relationship with Immune Cells

We used immunohistochemistry to validate HAMP expression in our clinical samples, and each sample was assessed three times. The antibody used to detect HAMP was the anti-hepcidin+hepcidin-2 antibody (EPR 18937, Abcam), and the details of the process are provided in the **Supplemental Materials and Methods**. Based on the results above, we calculated the positive cells% and H score to explore the relationship through immunohistochemistry. Positive cells%, which reflects the number of positive cells, was calculated as follows: number of positive cells/total number of cells. The H score is a histological scoring method for immunohistochemistry. The positive number and staining intensity in each section were converted into corresponding values to achieve semiquantitative staining of tissues. The H score was calculated as follows: H score = (PI × I) = (percentage of weak-intensity cells ×1)+(percentage of moderate-intensity cells ×2)+(percentage of strong-intensity cells ×3), where I represents the grade of positive cells. The scores were assigned as follows: negative without staining, 0 points; weak-positive light-yellow staining, 1 point; medium-positive brownish-yellow staining, 2 points; and strong-positive tan staining, 3 points. PI indicates the percentage of positive cells. We used three standard immunofluorescence assessments to validate the results.

### Biological Value of HAMP and the Prognostic Model

We used GSE14297 (21) to explore the potential value of HAMP in the liver metastasis of CRC, and we divided the data into three groups: tumor group, normal group and liver metastasis group. We downloaded the immunological therapy response data of CRC from The Cancer Imaging Archive (TCIA) database (https://tcia.at/) to explore the relationship between the expression of HAMP and therapeutic effects. We performed GSEA with gene sets that were downloaded from https://www.gsea-msigdb.org/gsea/msigdb to explore the biological functions of the hub genes and the risk score in the prognostic model. Then, the hub genes and risk score were divided into two groups: high- and low-expression groups based on the TCGA datasets. We used “c2.cp.kegg.v6.2.symbols.gmt” for analysis and selected the top five pathways. Then, we used the “plyr”, “ggplot2”, “grid”, and “gridExtra” R packages to integrate different significant pathways into a single diagram.

## Results

### Identification of Immune-Related Genes

Based on the dataset we downloaded from TCGA-COAD and TCGA-READ, we explored the DEGs (mRNA) between the control tissues and tumor tissues. We used immune genes and DEGs in the TCGA database to explore immune-related genes, including 472 genes.

### TF Regulatory Network and ceRNA Network

**Figure 1:**
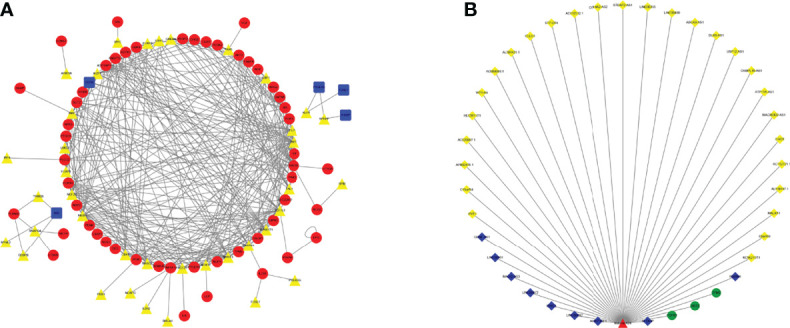
TF Regulatory Network and ceRNA Network. **(A)** TF network. The red circles represent high-risk IRGs, the blue squares represent low-risk IRGs, and the yellow triangles represent TFs. **(B)** ceRNA network based on prognosis-related genes. The light-yellow diamonds represent upregulated DElncRNAs, the blue diamonds represent downregulated DElncRNAs, the green circles represent mRNAs, and the red triangles represent DEmiRNAs.

We used univariate Cox regression analysis to explore survival-related genes (**Figure S1**) (**Supplementary File 1**). Based on the genes screened by univariate Cox analysis, we explored TF-related immune genes (PIRDEGs) and constructed a regulatory network between TFs and TF-related immune genes, as shown in **Figure 1A**. Based on the genes screened by uniCox analysis, we continued to explore hub prognostic genes through multivariate Cox analysis. Thirty-two genes were identified. We explored the regulatory relationships of these genes. There were 9 downregulated DElncRNAs, 27 upregulated DElncRNAs, 1 upregulated DEmiRNA, and 3 candidate target mRNAs (TPM2, AKT3, and FGFR1) in the network. Based on the DEmiRNA-DElncRNA and DEmiRNA-DEmRNA interactions, we built a DEmiRNA-DElncRNA-DEmRNA network (**Figure 1B**).

### Prognostic Model and Immune Infiltration and Risk Scores

**Figure 2:**
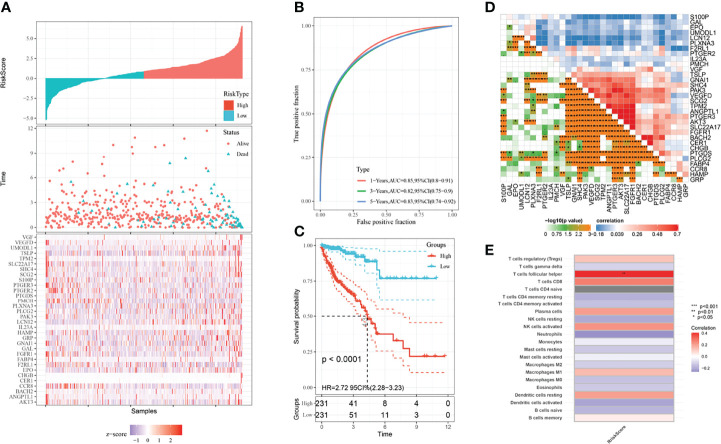
Prognostic model and immune infiltration. **(A)** Risk distribution of CRC patients. **(B)** ROC curves of CRC patients. The blue line represents the 5-year survival rate; the green line represents the 3-year survival rate; the red line represents the 1-year survival rate. **(C)** KM plots of the prognostic model. The blue line represents the low-risk group, and the red line represents the high-risk group. **(D)** The close relationship between the prognostic genes screened through multivariate Cox regression. **(E)** The relationship between 22 kinds of immune cells and the risk score of the prognostic model.

Based on the survival-related genes identified through multivariate Cox analysis, we calculated the prognostic risk score, and then, we divided the CRC patients into low- and high-risk groups to build a risk score model. The risk score distribution, ROC curve and Kaplan–Meier curves were analyzed and are shown in **Figures 2A–C**. The area under the curve (AUC) of the ROC curve was 0.85 for 1 year, 0.82 for 3 years and 0.83 for 5 years (**Figure 2B**). As shown in the Kaplan–Meier curves of the relationship of the risk score with overall survival (OS) in the low-risk and high-risk groups, the low-risk-score group had a stronger positive association with OS (P<0.0001; **Figure 2C**). We also explored their relationships with each other, and these genes had close relationships (**Figure 2D**). We analyzed the relationship with the risk score of the prognostic model and 22 kinds of immune cells: regulatory T cells, gamma delta T cells, follicular helper T cells, CD8 T cells, naive CD4 T cells, resting memory CD4 T cells, activated memory CD4 T cells, resting plasma cells, resting NK cells, NK-activated neutrophils, monocytes, resting mast cells, activated mast cells, M2 macrophages, M1 macrophages, M0 macrophages, eosinophils, resting dendritic cells, activated dendritic cells, naive B cells and memory B cells. As shown in **Figure 2E**, the risk score had a close correlation with follicular helper T cells (P<0.01) and had a small correlation with immune cells.

### TM Component and Score

Based on the datasets we downloaded, we estimated the immune and stromal component proportions in the TME through scoring. The ESTIMATE score is a combination of the immune score and stromal score. We analyzed the relationships of these scores with clinical characteristics, including age, sex, TNM classification and stage. For the ESTIMATE score, there was a significant difference between M0 and M1 macrophages. For the immune score, M0 and M1 macrophages also showed significant differences, and the fourth stage was different from the first to third stages. For the stomal score, there was a large difference between the N0 and N2 stages (**Figure S2**). We divided the scores into two groups: high-score and low-score groups. A high score indicates that there was a large number of immune or stromal components in the TME, and a low score indicates that there was a small number of immune or stromal components in the TME. We used Kaplan–Meier survival analysis to explore the relationship between the survival rate and TME scores. As shown in **Figure S3**, all the scores had little correlation with the survival time of the patients. We used CIBERSORT to explore the relative percentage of immune cells in CRC tumor tissue based on the TCGA database, and T cells and macrophages accounted for a large portion of immune cells in CRC (**Figure S4A**). Immunohistochemical results of two kinds of immune cells confirmed this conclusion (**Figure S4B-F**).

### Identification of DEGs in the TME

**Figure 3:**
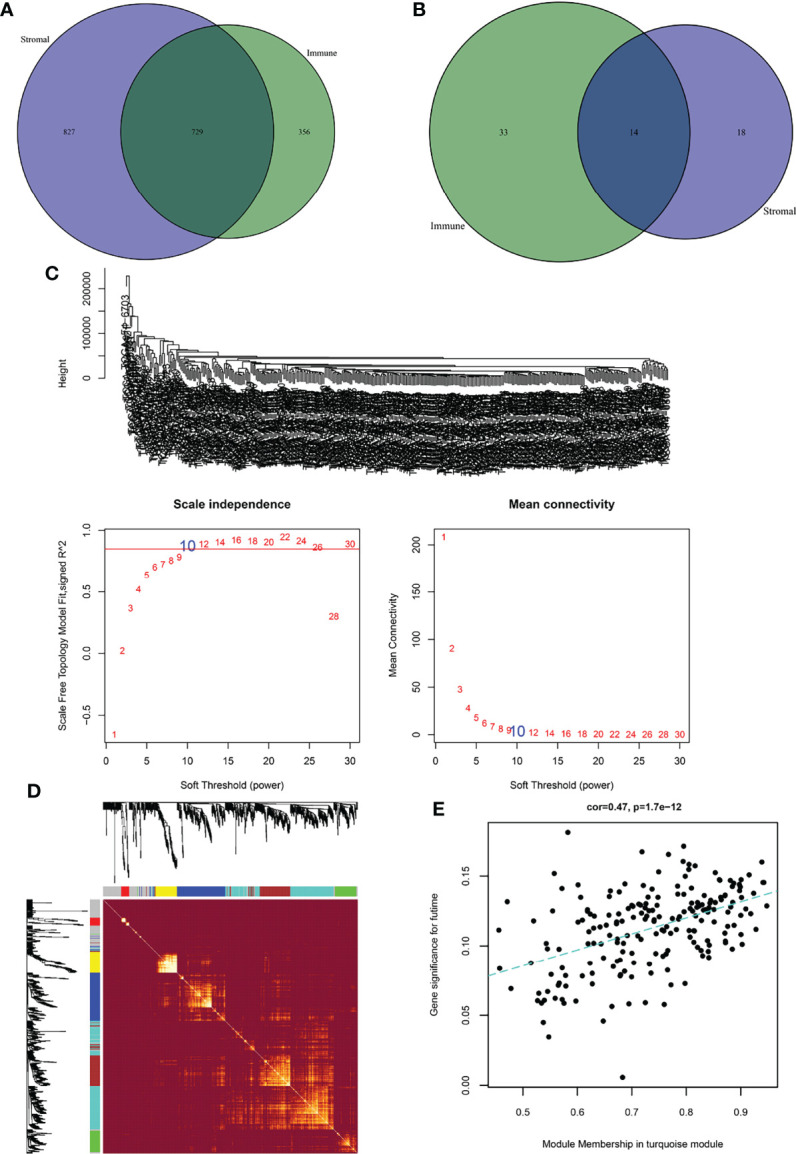
Identification of immune-related prognostic genes. **(A)** Upregulated genes between stromal and immune scores. **(B)** Downregulated genes between stromal and immune scores. **(C)** The upper figure shows the sample clustering result to determine whether there were outliers. The left figure is the soft threshold result, where the horizontal axis is the soft threshold (power), the vertical axis is the evaluation parameter of the scale-free network, and the soft threshold result is 10. The right figure shows the soft threshold and average connectivity. **(D)** TOM network heatmap of upregulated and downregulated genes. **(E)** Scatter plot of module eigengenes in the turquoise module.

To identify the DEGs in the TME, we first explored the DEGs in the stromal and immune components. As shown in **Figures 3A, B**, there were 14 genes in the low-score group and 729 genes in the high-score group of the stromal and immune groups. To further identify the key modules that were most correlated with CRC clinical traits, we performed WGCNA on the genes between the two groups. Clinical information such as age, TNM stage, and survival time was retrieved from TCGA. By setting a soft-thresholding power of 10, we eventually identified 7 modules (**Figures 3C, D**). From the heatmap of module-trait correlations, we found that the turquoise module, which contained 202 genes, was the most highly correlated with prognosis (P=1.7E-12; **Figure 3E**).

### GO and KEGG Enrichment Analyses of DEGs

**Figure 4:**
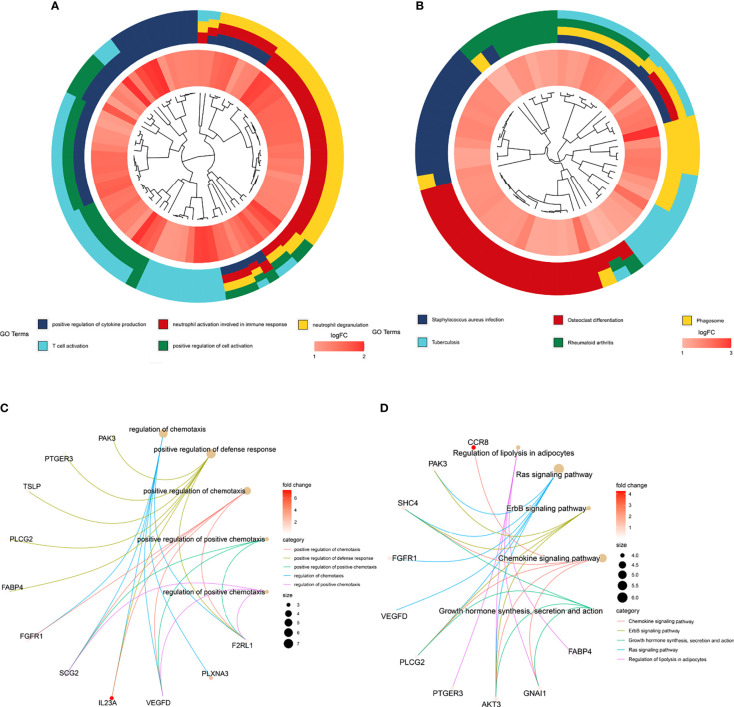
GO and KEGG enrichment of genes. **(A)** Top five GO enrichment terms of the turquoise module genes. **(B)** Top five KEGG enrichment pathways of the turquoise module genes. **(C)** Top five GO enrichment terms of the prognostic genes identified through multivariate Cox regression analysis. **(D)** Top five KEGG enrichment pathways of the prognostic genes identified through multivariate Cox regression analysis.

We explored the biological functions of the DEGs in the TME and the hub survival-related immune genes through GO and KEGG enrichment analyses following the criteria of P value <0.05 and adj P value<0.05. For the DEGs in the TME, the top five GO enrichment terms were positive regulation of cytokine production (P=2.13E-23), neutrophil activation involved in the immune response (P=5.16E-22), neutrophil degranulation (P=4.07E-12), positive regulation of cell activation (P=3.58E-20) and T-cell activation (P=2.68E-19) (**Figure 4A**). The top five KEGG enrichment pathways were *Staphylococcus aureus* infection (P=3.98E-21), osteoclast differentiation (P=6.46E-17), phagosome (P=2.83E-15), rheumatoid arthritis (P=1.45E-12) and tuberculosis (P=9.22E-12) (**Figure 4B**).

For hub survival-related immune genes, the top five terms in GO enrichment were regulation of chemotaxis (P=2.08E-06), positive regulation of defense response (P=3.02E-06), positive regulation of chemotaxis (P=4.12E-06), positive regulation of positive chemotaxis (P=9.94E-06) and regulation of positive chemotaxis (P=1.12E-05)) (**Figure 4C**); the top five enriched KEGG pathways were regulation of lipolysis in adipocytes (P=1.64E-05), Ras signaling pathway (P=3.46E-05), ErbB signaling pathway (P=8.57E-05), chemokine signaling pathway (P=0.0001683) and growth hormone synthesis, secretion and action (P=0.0003152) (**Figure 4D**).

### Clinical Significance and Correlation of TICs With HAMP

**Figure 5:**
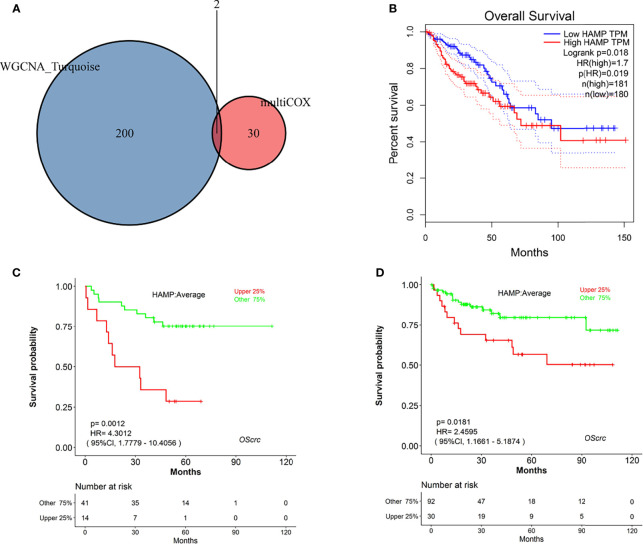
Identification of hub genes. **(A)** Venn diagram of genes commonly shared between the turquoise module and those screened by multivariate Cox analysis. **(B)** Kaplan–Meier plot of HAMP analysis by GEPIA online tools. **(C)** Kaplan–Meier plot of HAMP in GSE17537 analyzed by LOGpc online tools. **(D)** Kaplan–Meier plot of HAMP in GSE38832 analyzed by LOGpc online tools.

**Figure 6:**
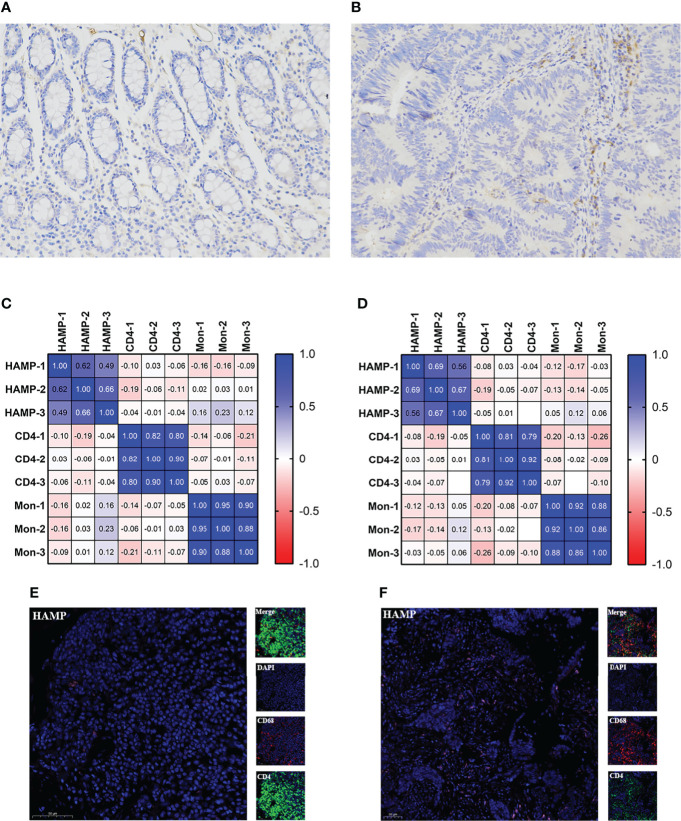
Relationship between HAMP and CD4+ T cells and M0 macrophages. **(A, B)** Immunohistochemistry of HAMP in clinical samples. The left figure is the control sample, and the right figure is the tumor sample. **(C)** Relationship of HAMP CD4+ T cells and M0 macrophages with positive cells. **(D)** Relationship of the H score of HAMP CD4+ T cells and M0 macrophages. **(E)** Immunofluorescence staining for the expression of CD4+ T cells (green), M0 macrophages (red) and HAMPs (pink) in control tissue. **(F)** Immunofluorescence staining of CD4+ T cells, M0 macrophages and HAMPs in tumor tissue. Scale bars=50 μm. CD4 represents CD4+ T cells, and Mon represents M0 macrophages.

Two genes, HAMP and CCR8, overlapped between the survival-related immune genes and the TME-related genes (**Figure 5A**), and we explored their prognostic value, as shown in **Figures 5B** and S5. As a result, only HAMP was associated with survival time. We validated the prognostic value in CRC in the GEO database (**Figures 5C, D**). We continued to explore the expression of HAMP between tumor tissue and paracarcinoma tissue in clinical specimens and public databases (**Figures 6A, B** and **S6**). The results indicated that HAMP is highly expressed in CRC tissue (P<0.001 in the public database). We also explored the clinical features related to this gene (**Figure S7**). There were no differences across the different stages and M grades. For the T stage, HAMP was significantly differentially expressed between stages T1 and T4 and stages T1 and T3; for the N stage, N0 and N2 showed large differences. Based on the CIBERSORT results, we assessed the relationship between the expression of HAMP and CD4^+^ T cells and M0 macrophages, the results by immunohistochemistry (**Figures 6C, D**) and three-standard immunofluorescence (**Figures 6E, F**) and showed that HAMP had a negative correlation with CD4^+^ T cells and M0 macrophages (**Supplementary Table**).

### Biological Functions of the Risk Score and HAMP

**Figure 7:**
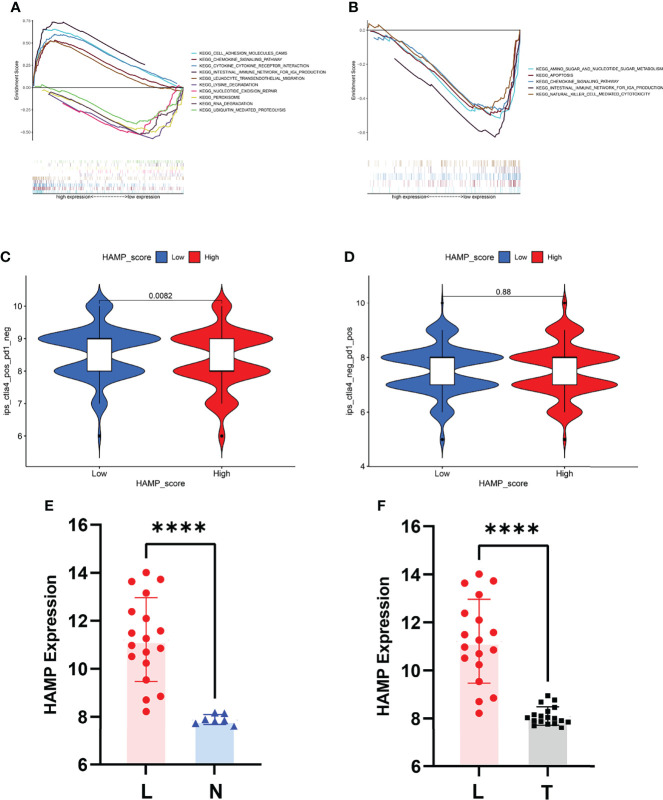
Biological value of HAMP and the risk score. **(A)** GSEA for the risk score. **(B)** GSEA for HAMP. **(C)** Relationship between the expression of HAMP and CTLA4-based immunotherapy. **(D)** Relationship between the expression of HAMP and PD1-based immunotherapy. **(E)** HAMP expression between the liver metastasis and normal groups in GSE14297. **(F)** HAMP expression between liver metastasis and tumor groups in GSE14297. L represents liver metastasis, N represents normal, and T represents tumor. ****P<0.0001.

We first performed GSEA to explore the biological functions of HAMP and the risk score of the prediction model in CRC in the TCGA-COAD and TCGA-READ datasets (**Figures 7A, B**). For the risk score of the prediction model, “amino sugar and nucleotide sugar metabolism”, “apoptosis”, “chemokine signaling pathway”, “intestinal immune network for IGA production” and “natural killer cell-mediated cytotoxicity” were enriched in the low-expression group, as shown in **Figure 7A**. The top 5 pathways with P<0.05 of the high and low HAMP expression groups are shown in **Figure 7B**. The high HAMP expression group was enriched in “cell adhesion molecules (CAMs)”, “chemokine signaling pathway”, “cytokine receptor interaction”, “intestinal immune network for IGA production” and “leukocyte transendothelial migration”. The low HAMP expression group was enriched in “lysine degradation”, “nucleotide excision repair”, “peroxisome”, “RNA degradation” and “ubiquitin-mediated proteolysis”. HAMP was also related to the effectiveness of CTLA4 immunotherapy, as shown in **Figures 7C, D**.

We also explored the value of HAMP in the liver metastasis of CRC. The P value between the liver metastasis and normal groups was <0.0001, and the P value between the liver metastasis and tumor groups was <0.0001 (**Figures 7E, F**).

## Discussion

CRC is a commonly diagnosed digestive cancer, and a large number of studies indicate that the TME of CRC tissue has an important influence on the occurrence and development of CRC (22, 23). The immune infiltration of TILs was also associated with the clinical outcome of CRC patients. Therefore, identifying a significant biomarker associated with the TME that has diagnostic value is necessary. Recently, gene microarrays have provided a way to discover novel biomarkers in public databases worldwide (24, 25). In our study, we first calculated the stromal score, immune score and ESTIMATE score of CRC tissue in the TCGA database. There was no significant difference between the TME scores, but based on the relative percentages of immune cells, the percentages of T cells and macrophages were high in CRC.

Then, we explored the key prognostic module between the high- and low-expression groups through WGCNA (26–28) and the turquoise module was the most associated with the prognosis of CRC. Among the significant genes, there are many genes that have been reported to be prognostic or diagnostic biomarkers for CRC, such as CCR8 (29), APOC1 (30), and CCL3 (31). We also explored the immune-related genes of CRC based on the DEGs between tumor and normal tissues in the TCGA database, and we explored the prognostic value of immune-related genes through univariate and multivariate Cox regression analyses.

TFs play an important role in the interpretation of the genome, regulate the expression of genes and are associated with immune responses (32). To explore the relationship between TFs and immune-related genes, we used immune genes screened by univariate Cox regression to construct a TF-immune-related gene regulatory network. Our network included 81 TFs, 70 high-risk immune-related genes and 7 low-risk immune-related genes, which may provide a new direction to study CRC immune mechanisms.

We performed GO and KEGG enrichment analyses to explore the biological functions of the genes screened by WGCNA and Cox regression. The top terms of GO analysis were all associated with immune regulation and function (33); the terms of KEGG analysis were associated with immune disease (34, 35) and immune regulation.

Based on the prognostic immune-related genes, we used Cox proportional hazards regression analysis to develop a prognostic risk score model. Then, we used ROC curves to validate the model and Kaplan–Meier risk survival analysis to explore the prognostic value of the model. The AUCs of the ROC curves, Kaplan–Meier plot and forest plot indicate that the prognostic risk model has high prognostic value and can predict the survival time well. We also explored the relationship between immune cell expression and the risk score model, which showed a close association.

Between the significant genes in the turquoise module of WGCNA and the immune-related genes screened by multivariate Cox regression, only HAMP and CCR8 were shared, and HAMP had high prognostic value for CRC. HAMP is a key negative regulator that maintains the balance of iron metabolism by downregulating serum iron levels and plays an important role in regulating the absorption, recycling and homeostasis of human iron (36, 37). HAMP regulation is balanced primarily by transferrin binding to iron (Tf) membrane iron transporter 1 (FP1) located on the cell surface. Iron modulin is also regulated by erythropoiesis, inflammation and other aspects. By binding with Tf, the only iron export protein in the cell, it induces its internalization and degradation, thus reducing the efflux of iron from the cell to the circulation and exerting its biological function (38, 39). Tumor cells need to store enough iron to meet their growth and development needs. Studies have revealed that HAMP is associated with CRC development and prognosis (40, 41). In our study, HAMP was highly expressed in tumor tissue, and we used CRC cells and tissue to validate its high expression in tumors. In addition, we explored its prognostic value and relationship with clinical information. We used online prognostic tools to explore the prognostic value in the GEO and TCGA databases, which showed that HAMP had good prognostic value for CRC. For clinical characteristics, HAMP showed no significant differences across the different stages, sexes, ages, or M stages of CRC but had a close relationship with stages N0-N2 and T1-T4, which may indicate that the expression of HAMP increases with the development of CRC. Based on the differential analysis between the liver metastasis and normal groups and between the tumor tissue and normal groups in the GEO database, HAMP was significantly different.

To further explore its immune characteristics, we used vioplot to show the relationship between the expression of immune cells and HAMP. The results showed that B cells, CD4^+^ T cells and gamma T cells were closely associated with HAMP. Based on the CIBERSORT results, we explored the relationship between the expression of HAMP and CD4^+^ T cells and M0 macrophages. The negative correlation and characteristic expression of HAMP in CRC tissue may provide a new perspective for exploring the immune infiltration of CRC. We also explored the biological functions of HAMP and the risk score model. The GSEA results indicated that HAMP was enriched in “cell adhesion molecules (CAMs)”, “chemokine signaling pathway”, “cytokine receptor interaction”, “intestinal immune network for IGA production” and “leukocyte transendothelial migration” in the low-expression group and “lysine degradation”, “nucleotide excision repair”, “peroxisome”, “RNA degradation” and “ubiquitin mediated proteolysis” in the high-expression group, and the risk score model was enriched in “amino sugar and nucleotide sugar metabolism”, “apoptosis”, “chemokine signaling pathway”, “intestinal immune network for IGA production” and “natural killer cell-mediated cytotoxicity”. The GSEA results suggested that HAMP and the risk score model have a close relationship with immune regulation and may influence CRC development and progression (42, 43).

In summary, HAMP can serve as a prognostic biomarker for CRC and has a close relationship with immune cell expression. We also constructed a risk score model for predicting OS in CRC. Our study provides a new direction for immunotherapy treatment strategies and predicts the prognosis of CRC. However, our work has some limitations. In the future, we will further verify the expression of HAMP and develop our model in a large sample cohort and further explore the regulatory mechanism between HAMP and immune cells and CTLA4 immunotherapy.

## Data Availability Statement

The datasets presented in this study can be found in online repositories. The names of the repository/repositories and accession number(s) can be found in the article/**Supplementary Material**.

## Ethics Statement

The studies involving human participants were reviewed and approved by National Cancer Center/National Clinical Research Center for Cancer/Cancer Hospital. The patients/participants provided their written informed consent to participate in this study.

## Author Contributions

F-ZW designed the research; Z-JW, S-WM, J-L and Y-BM organized the data; J-NC, F-QZ and WZ analyzed and visualized the data; F-ZW drafted the article; and WY and QL revised the paper. All authors contributed to the article and approved the submitted version.

## Funding

Key Project of National Key R & D Plan “Research on Prevention and Control of Major Chronic Non-Communicable Diseases” (No. 2019YFC1315705), National Natural Science Foundation of China (Grant No 51972343 and Grant No 51937011), the China Cancer Foundation Beijing Hope Marathon Special Fund (No. LC2017L07), and the Medical and Health Science and Technology Innovation Project of the Chinese Academy of Medical Sciences (No. 2017-12M-1-006) of China.

## Conflict of Interest

The authors declare that the research was conducted in the absence of any commercial or financial relationships that could be construed as a potential conflict of interest.

## Publisher’s Note

All claims expressed in this article are solely those of the authors and do not necessarily represent those of their affiliated organizations, or those of the publisher, the editors and the reviewers. Any product that may be evaluated in this article, or claim that may be made by its manufacturer, is not guaranteed or endorsed by the publisher.

## Abbreviations

GEO, Gene Expression Omnibus; TCGA, The Cancer Genome Atlas; GO, Gene Ontology; KEGG, Kyoto Encyclopedia of Genes and Genomes; WGCNA, Weighted Gene Coexpression Network Analysis, RRA: Robust Rank Aggregation; DEG, Differentially Expressed Gene; TNM, Tumor Node Metastasis; GSEA, Gene Set Enrichment Analysis; MCC, Maximal Clique Centrality; ROC, Receiver Operating Characteristic.
