# Supplementary material for: HAMP as a Prognostic Biomarker for Colorectal Cancer Based on Tumor Microenvironment Analysis
Source: Front Oncol. 2022 Aug 5;12:884474. doi: 10.3389/fonc.2022.884474 (PMC9386429; doi:10.3389/fonc.2022.884474)
Supplement: Supplementary file 1 [file DataSheet_1.docx]

**Supplemental Materials and Methods**

**Procedure of paraffin section immunohistochemistry experiment：**

1. Deparaffinzing and rehydrating the paraffin section: the sections were washed in xylene (Chemical Reagent Co.LTD) I for 15min - xylene II for 15min - xylene III for 15min- pure ethanol(Chemical Reagent Co.LTD) I for 5min - pure ethanol II for 5min - 85% alcohol for 5min - 75% alcohol for 5min -Wash in distilled water.
2. Antigen retrieval: Tissue sections were immerse in EDTA repair buffer (PH9.0) (G1203,Servicebio) for antigen repair in the microwave oven, with a medium heat for 8min to boiling, a cease-fire for 8min for heat preservation, and then a medium and low heat for 10min. After natural cooling, the slides were placed in PBS (PH7.4)(Servicebio,G0002) and shaken on a decolorizing shaker for 3 times, 5min each.
3. Block endogenous peroxidase: The slides were placed in 3% hydrogen peroxide(Chemical Reagent Co.LTD, 10011208) solution and incubated at room temperature, dark for 25 min. The slides were placed in PBS (PH7.4)(Servicebio,G0002) and washed by shaking on a decolorizing shaker(Servicebio,TSY-B) for 3 times, 5min each.
4. Serum sealing: 3%BSA(Servicebio,G5001) was added to the circle to evenly cover the tissue, and the tissues are sealed for 30 minutes at room temperature.

5. Primary antibody incubation: the sealing solution is gently removed, the primary antibody prepared with PBS in a certain proportion is added to the sections, and the sections are placed flat in a wet box and incubated overnight at 4℃. (HAMP: PBS 1:100; CD4:PBS,1:200; CD68:PBS,1:400)

6. Secondary antibody incubation: the sections are placed in PBS(PH7.4) and washed by shaking on the decolorizing shaker 3 times for 5 minutes each. After the sections are slightly shaken and dried, the tissues are covered with secondary antibody (HRP labeled Goat anti-rabbit) and incubated at room temperature for 50 minutes.

7. DAB chromogenic reaction: the sections are placed in PBS(PH7.4) and shaken on the decoloring shaker 3 times for 5 minutes each. DAB(Servicebio,G1211) color developing solution newly prepared is added in the circle after the sections are slightly dried. The color developing time is controlled under the microscope. The positive is brownish yellow.Rinse the sections with tap water to stop the reaction.

8. Nucleus counterstaining: the sections are counterstained with hematoxylin stain solution(Servicebio,G1004) for about 3 minutes; washed with tap water; differentiated with hematoxylin differentiation solution(Servicebio,G1309) for several seconds; washed with tap water; treated with hematoxylin returning blue solution(Servicebio,G1340); washed with running water.

9. Dehydration and mounting: place the section in 75% alcohol for 5 minutes - 85% alcohol for 5 minutes - pure ethanol Ⅰ for 5 minutes - pure ethanol Ⅱ for 5 minutes - xylene Ⅰ 5 minutes, dehydrated and transparent, remove the sections from xylene and let them dry slightly, then mount the sections with neutral gum.

10. Visualize staining of tissue under a microscope, acquisitive and analysis image.

Ⅲ Interpretation of the immunohistochemical results of paraffin section

The nucleus of hematoxylin stained is blue, and the positive expression of DAB is brownish yellow.

**Procedure of Three-standard immunofluorescence：**

1. Deparaffinize and rehydrate: incubate sections in 2 changes of xylene, 15 min each. Dehydratein 2 changes of pure ethanol for 5 min, followed by dehydrate in gradient ethanol of 85% and 75% ethanol (SCRC, 100092183), respectively for 5 min each. Wash in distilled water.
2. Antigen retrieval: immerse the slides in EDTA antigen retrieval buffer (pH 8.0). Maintain at sub-boiling temperature for 8 min - standing for 8 min - sub-boiling temperature for 7 min - air cooling. Wash threetimes with PBS (pH 7.4) in a Rocker device for 5 min each.
3. Circle, Block endogenous peroxidase: wash three times with PBS (pH 7.4) in a Rocker device, 5 min each eliminate obvious liquid and mark the objective tissue. Immerse in 3% H_2_O_2_ and incubate at room temperature for 15 min then kept in dark place. Wash three times with PBS (pH7.4) in a Rocker device for 5 min each samples.
4. Block with serum: dry and mark the objective tissue. Cover objective tissues with 10% donkey serum at room temperature for 30 min.
5. First primary antibody: throw away the blocking solution slightly. Incubate slides with the first primary antibody overnight at 4℃.
6. Corresponding secondary antibody marked with HRP: wash slides three times with PBS (pH7.4) in a Rocker device, 5 min each. Then throw away liquid slightly. Cover objective tissue with secondary antibody, then incubate at room temperature for 50 min in dark condition.
7. CY3-TSA solution: wash slides three times with PBS (pH 7.4) in a Rocker device for 5 min each.Incubate slides with CY3-TSA solution for 10 min in dark condition then wash slides three times for 5min each with TBST in a Rocker device.
8. Microwave treatment: immerse the slides in EDTA antigen retrieval buffer (pH8.0) and maintain at a sub-boiling temperature for 8 min - standing for 8 min - sub-boiling temperature for 7 min - remove the primary antibodies and Secondary antibodies combined in tissue.
9. Add second primary antibody: Incubate slides with primary antibody overnight at 4 ℃.
10. Second corresponding secondary antibody marked with HRP: wash slides three times with PBS (pH 7.4) in a Rocker device for 5 min each. Then throw away liquid slightly and cover objective tissue with secondary antibody, incubate at room temperature for 50 min in dark condition.
11. Add FITC-TSA: wash slides three times with PBS (pH 7.4) in a Rocker device, 5 min each. Incubate slides with FITC-TSA solution for 10 min in dark condition.After incubate, wash slides for three times with TBST in a Rocker device for 5min each.
12. Microwave treatment: immerse the slides in EDTA antigen retrieval buffer (pH 8.0) at a sub-boiling temperature for 8 min, standing for 8 min another sub-boiling temperature for 7 min and remove the primary antibodies and Secondary antibodie scombined in tissues.
13. Incubate the third primary antibody: Incubate slides with primary antibody overnight at 4 ℃
14. Add the secondary antibody marked with CY5: wash slides three times used PBS (pH 7.4) in a Rocker device, 5 min each. Throw away liquid slightly and cover objective tissue with secondary antibody incubate at room temperature for 50 min in dark condition.
15. DAPI counterstain in nucleus: incubate with DAPI solution at room temperature kept in dark condition for 10 min,.
16. Spontaneouss fluorescence quenching: wash slides three times with PBS (pH 7.4) in a Rocker device, 5 min each. Dry slides and incubate slides with spontaneouss fluorescence quenchingre agent for 5 min. Wash slides with flowing water for 10 min.
17. Mount: wash three times with PBS (pH 7.4) in a Rocker device, 5 min each. Dry slides slightly and cover slides with anti-fademounting medium.
18. Detection in slice scanner: for DAPI glows blue by UV excitation wave length 330-380nm and emission wave length 420nm; for FITC glows green by excitation wave length 465-495nm and emission wave length 515-555nm; for CY3 glows red by excitation wave length 510-560nm and emission wave length 590nm. CY5 glows pink by excitation wave length 608-648nm and emission wavelength 672-712nm. (CY5 was originally red, in order to distinguish it from CY3, we set it to pink light.)
