# Supplementary figures and images for: HAMP as a Prognostic Biomarker for Colorectal Cancer Based on Tumor Microenvironment Analysis
Source: Front Oncol. 2022 Aug 5;12:884474. doi: 10.3389/fonc.2022.884474 (PMC9386429; doi:10.3389/fonc.2022.884474)

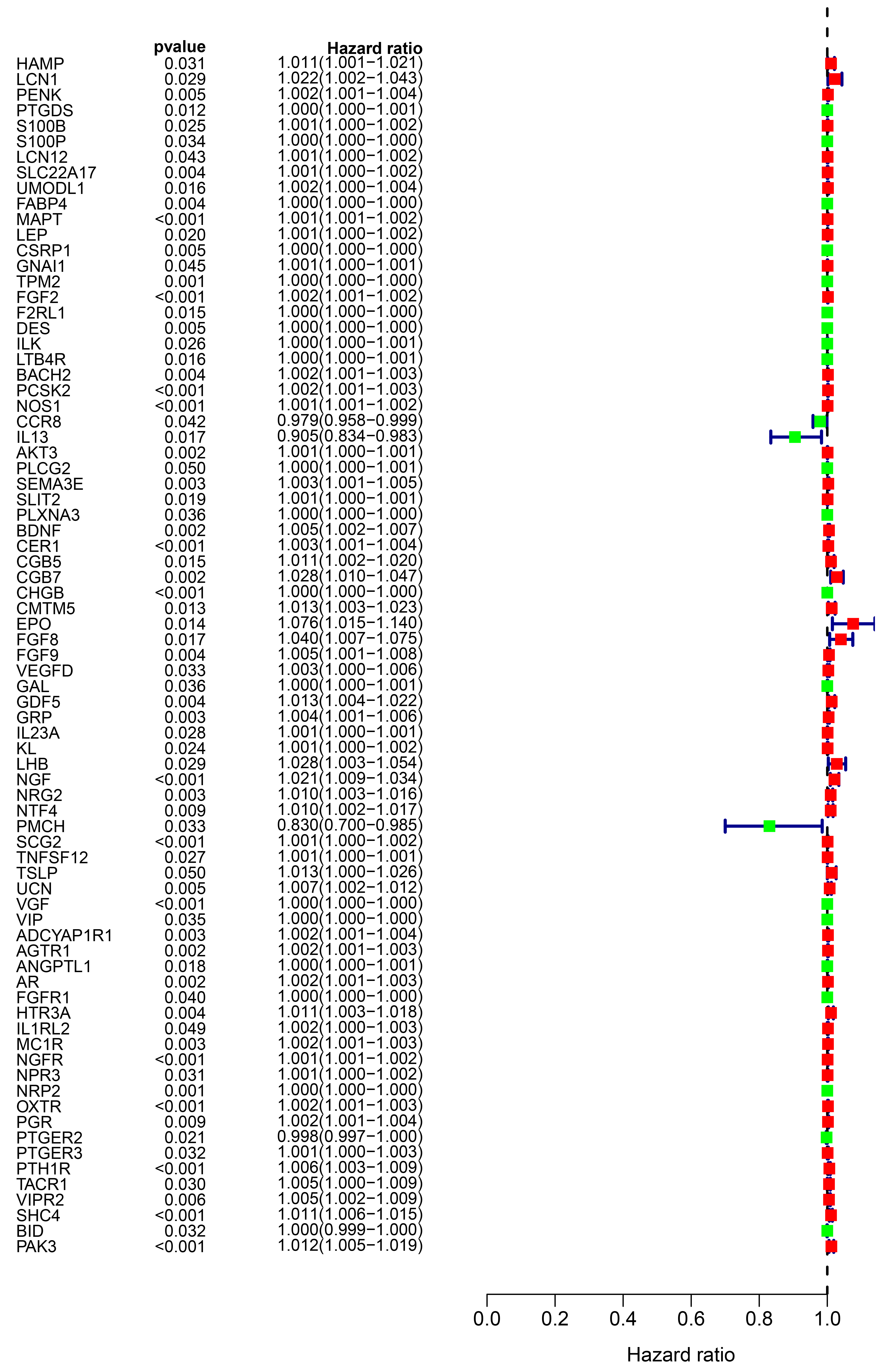

Supplement: Supplementary file 2 [file Image_1.tif]

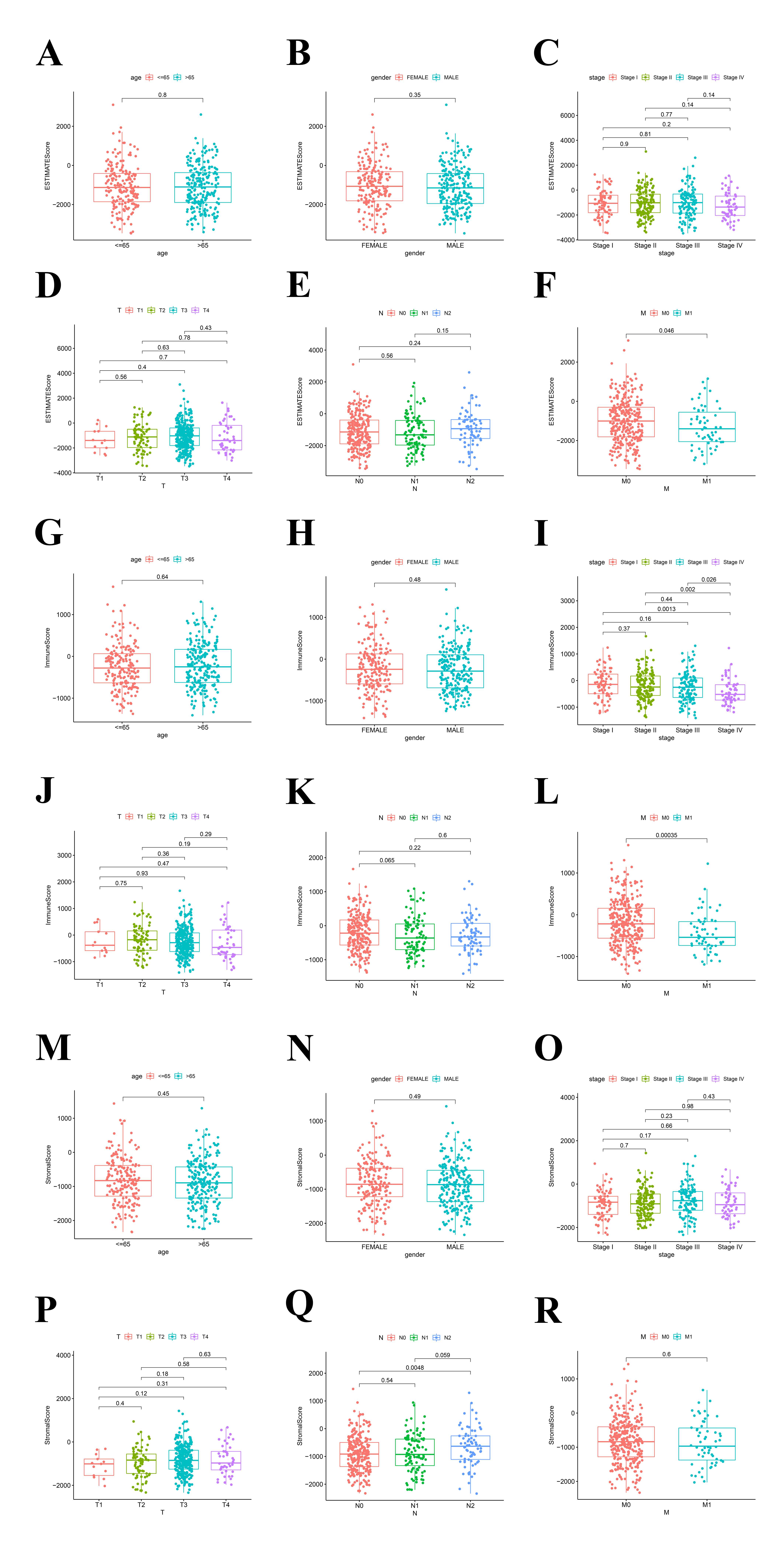

Supplement: Supplementary file 3 [file Image_2.tif]

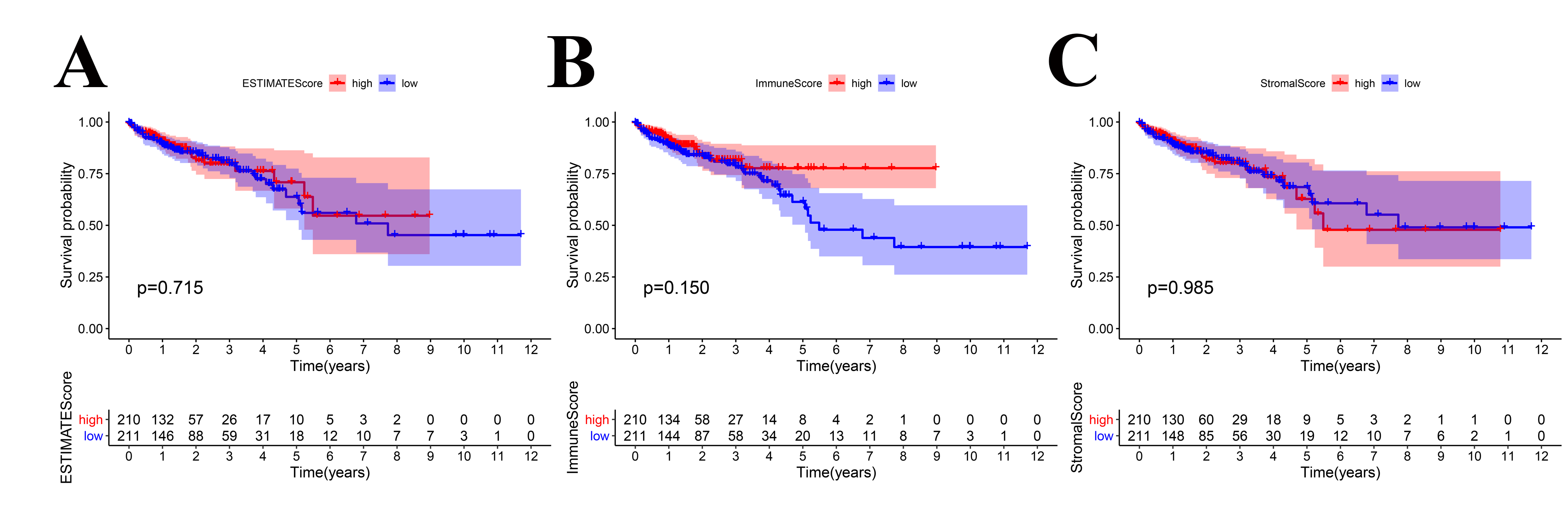

Supplement: Supplementary file 4 [file Image_3.tif]

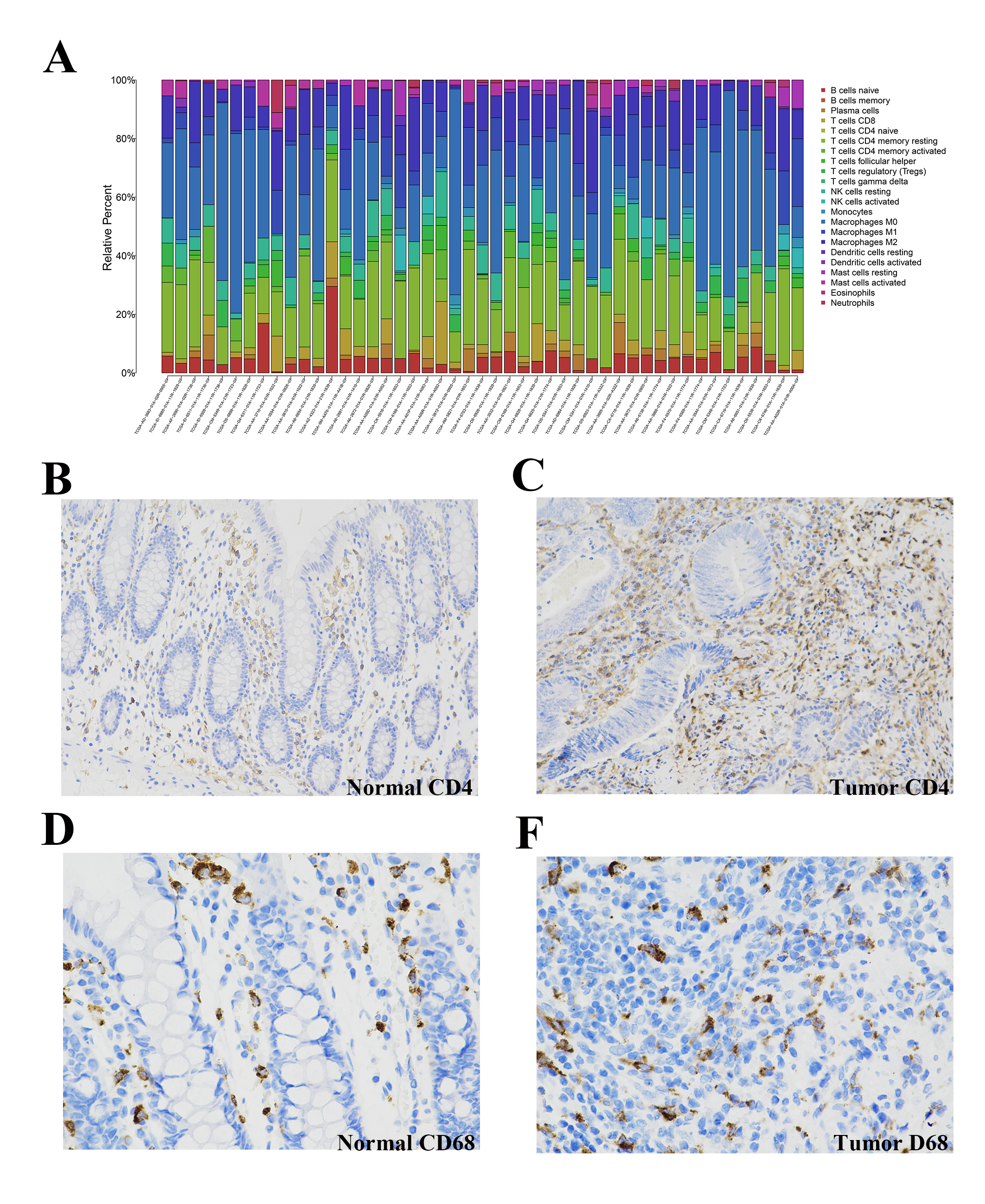

Supplement: Supplementary file 5 [file Image_4.tif]

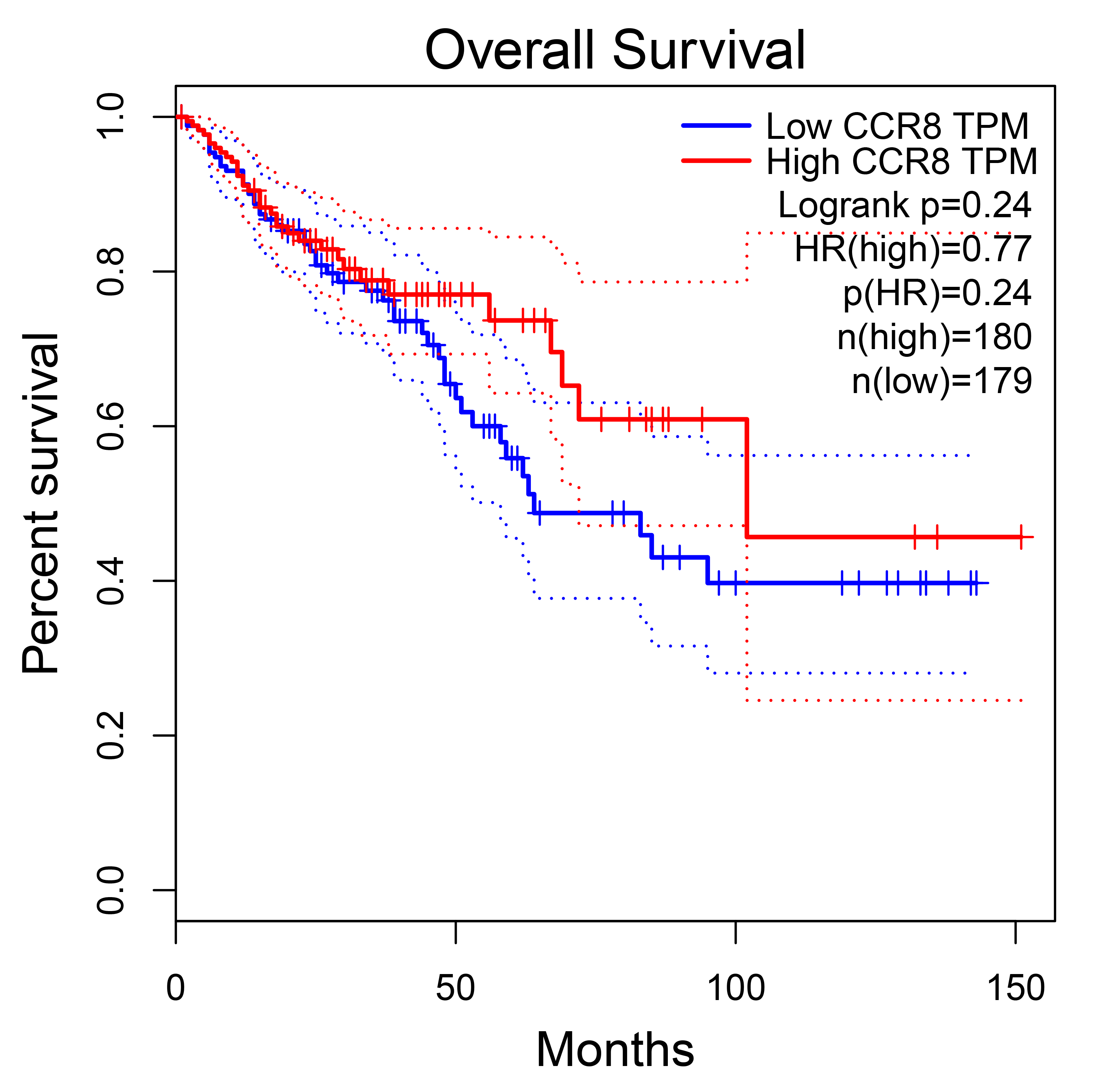

Supplement: Supplementary file 6 [file Image_5.tif]

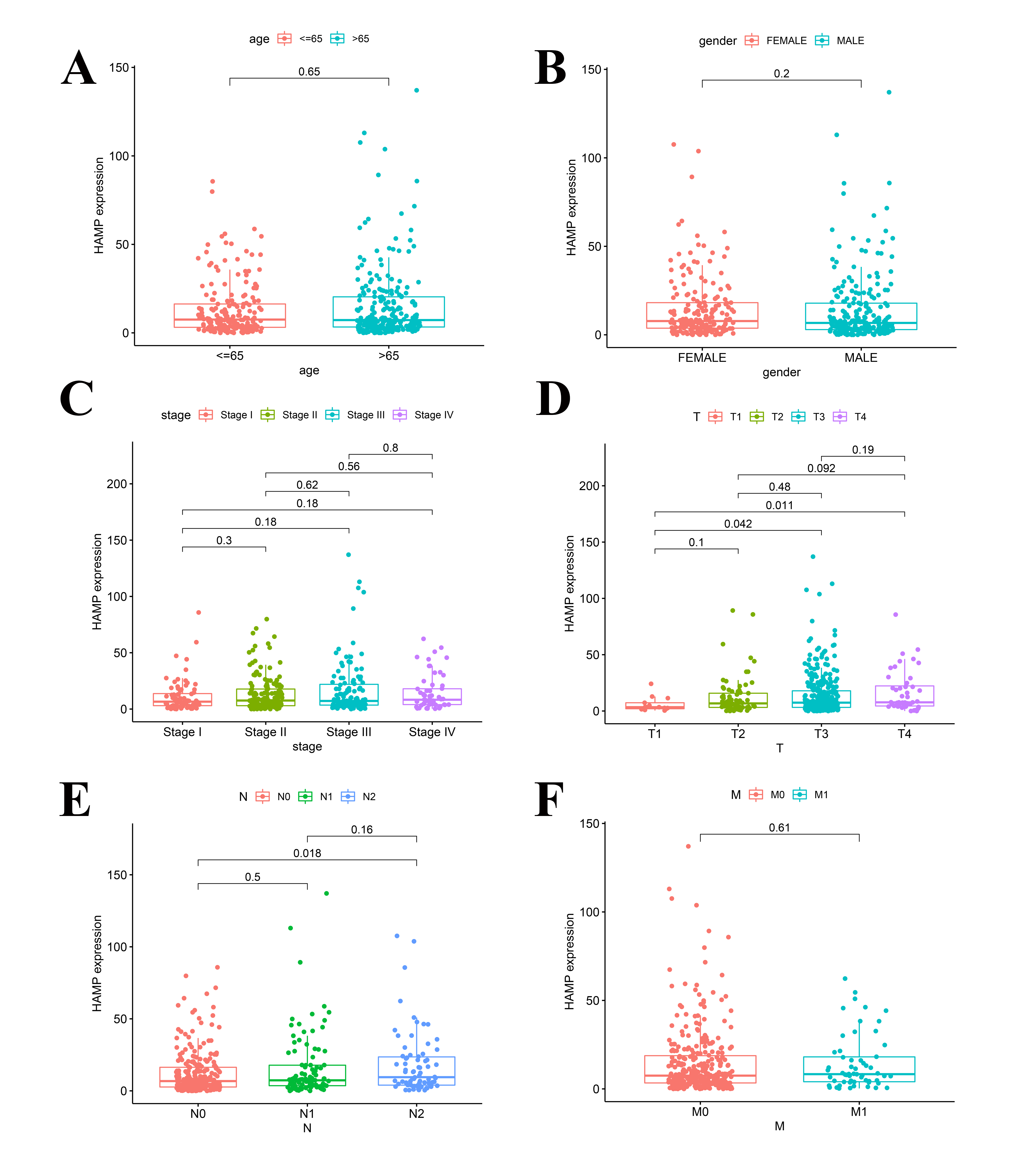

Supplement: Supplementary file 7 [file Image_6.tif]
